# Opposing Functions of Classic and Novel IL-1 Family Members in Gut Health and Disease

**DOI:** 10.3389/fimmu.2013.00181

**Published:** 2013-07-09

**Authors:** Loris R. Lopetuso, Saleem Chowdhry, Theresa T. Pizarro

**Affiliations:** ^1^Department of Pathology, Case Western Reserve University School of Medicine, Cleveland, OH, USA; ^2^Internal Medicine, Gastroenterology Division, Catholic University of Rome, Rome, Italy; ^3^Digestive Health Institute, University Hospitals, Cleveland, OH, USA

**Keywords:** IL-1 family of cytokines, inflammatory bowel disease, colitis, inflammation-associated colon cancer, intestinal homeostasis, Toll/IL-1 receptor family, mucosal wound healing, intestinal fibrosis

## Abstract

In addition to their well-established role(s) in the pathogenesis of gastrointestinal (GI)-related inflammatory disorders, including inflammatory bowel disease (IBD) and inflammation-associated colorectal cancer (CRC), emerging evidence confirms the critical involvement of the interleukin-1 (IL-1) cytokine family and their ligands in the maintenance of normal gut homeostasis. In fact, the paradigm that IBD occurs in two distinct phases is substantiated by the observation that classic IL-1 family members, such as IL-1, the IL-1 receptor antagonist (IL-1Ra), and IL-18, possess dichotomous functions depending on the phase of disease, as well as on their role in initiating vs. sustaining chronic gut inflammation. Another recently characterized IL-1 family member, IL-33, also possesses dual functions in the gut. IL-33 is upregulated in IBD and potently induces Th2 immune responses, while also amplifying Th1-mediated inflammation. Neutralization studies in acute colitis models, however, have yielded controversial results and recent reports suggest a protective role of IL-33 in epithelial regeneration and mucosal wound healing. Finally, although little is currently known regarding the potential contribution of IL-36 family members in GI inflammation/homeostasis, another IL-1 family member, IL-37, is emerging as a potent anti-inflammatory cytokine with the ability to down-regulate colitis. This new body of information has important translational implications for both the prevention and treatment of patients suffering from IBD and inflammation-associated CRC.

## Background and Introduction

### Structure and function of the intestinal gut mucosa

The gastrointestinal (GI) tract, with its epithelial barrier consisting of a total surface area of approximately 200 m^2^, is man’s most widely exposed organ system to the external environment. The intestinal barrier represents a functional unit responsible for two main tasks crucial for survival of the individual: allowing nutrient absorption, and defending the body from penetration of unwanted, often dangerous, macromolecules. In fact, the gut mucosa is a multi-layered system consisting of an external “anatomical” barrier and an inner “functional” immunological barrier. Commensal gut microbiota, a mucous layer, and the intestinal epithelial monolayer constitute the anatomical barrier. The deeper, inner layer consists of a complex network of immune cells organized in a specialized and compartmentalized system known as gut-associated lymphoid tissue or GALT. GALT represents both isolated and aggregated lymphoid follicles and is one of the largest lymphoid organs, containing up to 70% of the body’s total number of immunocytes, and is involved in responding to pathogenic microorganisms and providing immune tolerance to commensal bacteria. The ability of GALT to interact with luminal antigens rests on specific mucosal immune cells (i.e., dendritic cells and M-cells), primarily localized to Peyer’s patches within the ileum, that are intimately positioned at the mucosal-environmental interface and internalize microorganisms and macromolecules. These specialized immune cells have the ability to present antigen to naïve T-lymphocytes, which subsequently produce cytokines and activate mucosal immune responses, when needed. From the intracellular point of view, inflammasomes are a group of protein complexes that assemble upon recognition of a diverse set of noxious stimuli and are now considered the cornerstone of the intracellular surveillance system. They are able to sense both microbial and damage-associated molecular patterns (DAMPs) and initiate a potent innate, anti-microbial immune response (1). The interaction of these components sustains the maintenance of the delicate equilibrium needed for intestinal homeostasis. Many factors can alter this balance, including alterations in the gut microflora, modifications of the mucus layer, and epithelial damage, leading to increased intestinal permeability and translocation of luminal contents to the underlying mucosa (2). The integrity of these structures is necessary for the maintenance of normal intestinal barrier function. Dysregulation of any of the aforementioned components have been implicated not only in the pathogenesis of inflammatory bowel disease (IBD), but many other GI disorders, including infectious enterocolitis, irritable bowel syndrome, small intestinal bowel overgrowth, and allergic food intolerance (3–5).

### The Toll/IL-1 receptor superfamily in the GI tract

The role of the Toll/IL-1 Receptor (TIR) superfamily and their respective ligands, of which interleukin-1 (IL-1)-like molecules belong, is well established in the pathogenesis of several autoinflammatory and chronic immune disorders (6). However, the emerging concept that Toll-like receptors (TLRs), as well as IL-1 and its related cytokine family members, also play a critical role in health and the maintenance of immune homeostasis is gaining increasing acceptance. The GI system, in fact, represents one of the best examples of where these opposing mechanisms simultaneously take place (7). A large body of literature exists that support the contribution of various TLRs and IL-1 family members, particularly IL-1 and IL-18, to the pathogenesis of IBD, such as Crohn’s disease (CD) and ulcerative colitis (UC), as well as GI-related cancers. However, while selective blockade of pro-inflammatory cytokines is one of the most effective strategies to down-regulate mucosal inflammation in IBD (8), Phase I clinical trials using strategies to neutralize either IL-1 or IL-18 have failed to show significant efficacy in treating patients with UC and CD, respectively. One potential cause for this failure is the dichotomous functions of these IL-1 family members in inducing disease pathogenesis, while simultaneously promoting protection, within the intestinal gut mucosa.

In fact, new insights into the role of cytokine-driven pathways in mucosal immunity have been described based on several recent studies in animal models of acute intestinal injury, repair, and chronic inflammation. Information derived from these studies reveal that intestinal homeostasis and inflammation are driven by cellular elements and soluble mediators that mediate both processes, with several cytokines exhibiting opposing roles, depending upon the specific setting. This concept is most strongly supported by members of the IL-1 family of cytokines in the pathogenesis of IBD (Table 1) (9–22), where the same cytokine can possess both classic pro-inflammatory properties, as well as protective, anti-inflammatory functions, which is primarily dependent on the presence of receptor-bearing cells during the host’s disease state. Related to this notion is the dogma that chronic intestinal inflammation characteristic of IBD develops through two distinct phases (21). Early disease refers to the initial events that take place when homeostatic mechanisms initially fail and acute inflammatory responses cannot be resolved. In contrast, late disease refers to the period when adaptive immunity has been irreversibly primed toward a specific effector phenotype. During these distinct stages of disease progression, innate cytokines play diverse, and often times, dichotomous roles (21).

**Table 1:** **Role of IL-1 cytokine family members in IBD and in GI-related cancers**.

Common name	IL-1 family name	Ligand-binding chain	Disease association	Potential role in IBD	Potential role in Gl-related cancers
IL-lα	IL-1F1	IL-lR type I	CD, UC	Protective during early phase of inflammation	Induction of tumor growth, metastasis formation, and angiogenesis in gastric, liver, colon, and pancreatic cancer
IL-1β	IL-1F2	IL-lR type I	CD, UC	Protective during early phase of inflammation	Induction of tumor growth, metastasis formation, and angiogenesis in gastric, liver, colon, and pancreatic cancer
IL-IRa	IL-1F3	IL-IR type I	UC	Potential dual role	Protective
IL-18	IL-1F4	IL-18Rα	CD	Protective during early phase of inflammation	Protective in inflammation-associated colon cancer
IL-36Ra	IL-1F5	IL-lRrp2	Unknown	Unknown	Unknown
IL-36α	IL-1F6	IL-lRrp2	Unknown	Unknown	Unknown
IL-37	IL-1F7	IL-18Ra	Unknown for human IBD, antagonist for DSS colitis	Protective (correlates with breakdown of intestinal barrier)	Expressed in colon cancer cells
IL-36β	IL-1F8	IL-lRrp2	Unknown	Unknown	Unknown
IL-36γ	IL-1F9	IL-lRrp2	Unknown	Unknown	Unknown
IL-38	IL-1F10	IL-lRrp2	Unknown	Unknown	Unknown
IL-33	IL-1F11	ST2	UC	Protective	Possible support of tumor formation and progression

As such, aside from the established pro-inflammatory properties of IL-1α, IL-1β, IL-18, and their downstream signaling molecules shared with TLR family members, such as nuclear factor kappa-light-chain-enhancer of activated B cells (NF-κB) and myeloid differentiation primary response 88 (MyD88), a growing body of evidence indicates that these mediators are necessary for the maintenance of mucosal homeostasis by effectively handling microbiota, as well as by protecting and restoring the integrity of the epithelial barrier (23–25). While little is known regarding the potential contributions of other IL-1 family members, such as IL-36, IL-36Ra, IL-37, and IL-38, in chronic intestinal inflammation and gut health, the evolving literature regarding the role of IL-33, the most recently described IL-1 family member is, at present, ambiguous and may reflect yet another example of an innate-type cytokine that possesses multiple functions depending on the immunological status and genetic susceptibility of the host. Although one of the first observations of IL-33-dependent functions in the gut was potent epithelial proliferation and mucus production (26), suggesting the promotion of mucosal repair and healing, dysregulated or uncontrolled IL-33 production may also lead to more pathogenic features characteristic of IBD, including epithelial barrier dysfunction, chronic, relapsing inflammation, and formation of fibrotic lesions (27, 28).

In the present review, we will comprehensively evaluate the role of IL-1 family members and their associated ligands in modulating mucosal homeostasis and chronic inflammation within the GI tract, as well as touch on the potential contribution of these important receptor-ligand pairings to GI tumorigenesis and cancer. Moreover, we speculate about the potential implications of the opposing functions of IL-1 family members for treating chronic intestinal inflammation and inflammation-associated colorectal cancer (CRC), as well as in designing more efficacious strategies for the prevention and treatment of these devastating GI pathologies.

## Pathogenic Role of Classic IL-1 Family Members in Chronic Intestinal Inflammation and Inflammation-Associated CRC

### Pathogenic effects of IL-1α, IL-1β, and IL-1Ra during chronic intestinal inflammation

**Figure 1:**
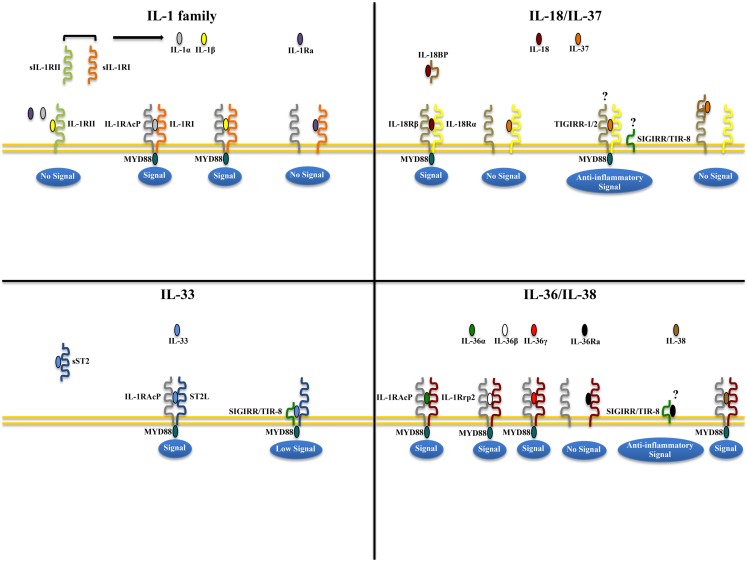
**Receptor-ligand pairing of IL-1 family members**. Productive pairings of ligand, binding receptor, and accessory protein for the IL-1 (upper left), IL-18/IL-37 (upper right), IL-33 (lower left), and IL-36/IL-38 (lower right) systems. The overall bioactivity of IL-1 family agonists is dependent on the prevalent isoform and receptor binding domain/accessory protein present on effector cells. Promiscuous receptor/co-receptor binding of agonists and antagonists imply that IL-1 family members cannot be considered in isolation, but in the context of other IL-1 family members that can influence their overall integrative effects and impact on disease pathogenesis.

**Figure 2:**
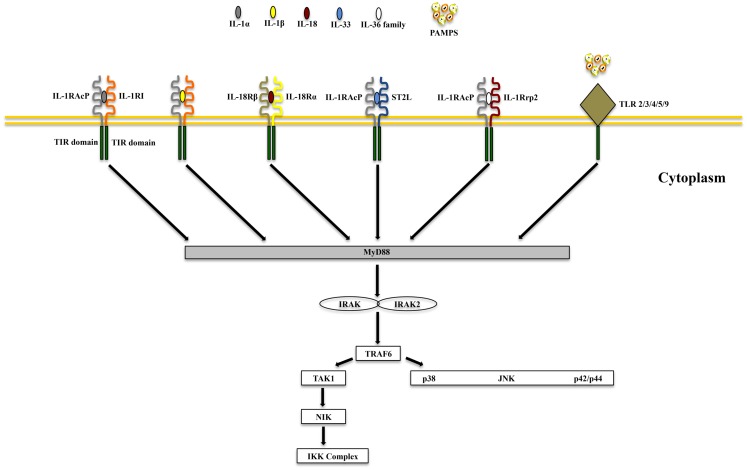
**Common signaling pathway for Toll/IL-1R (TIR) family members**. TLRs possess significant homology to IL-1R family members and share a similar cytoplasmic TIR domain with receptors of IL-1, IL-18, IL-33, and IL-36. The TIR domain is essential for recruitment of cytoplasmic adapter proteins, which in turn initiates downstream signaling cascades. PAMPS, pathogen-associated molecular patterns.

IL-1α and IL-1β (IL-1F1 and F2, respectively) are derived from different genes, but are functionally similar, and both bind to the IL-1R type I (IL-1RI) (Figure 1). This is followed by recruitment of the co-receptor chain, IL-1R accessory protein (IL-1RAcP), and a receptor complex is formed. The IL-1R complex can then recruit the adaptor protein, MyD88, to the TIR domain, after which several kinases are phosphorylated, NF-κB translocates to the nucleus, and the transcription of several inflammatory genes takes place (Figure 2). Although they exhibit similar biological activities, IL-1α and IL-1β differ in the manner in which they are processed and secreted. IL-1α is localized in the cytosol or cell membrane and is believed to regulate the intracellular environment (29), but can also be secreted into the extracellular compartment and serve as a soluble mediator (30). In contrast, IL-1β is first cleaved to its mature active form and then secreted extracellularly. Patients with infectious or inflammatory conditions exhibit elevated plasma concentrations of IL-1β but not IL-1α, suggesting a systemic role for IL-1β (30). With the sole exception of IL-1 receptor antagonist (IL-1Ra), each member of the IL-1 family is first synthesized as a precursor molecule without a clear signal peptide for processing and secretion. IL-1α, similar to the newest member of the IL-1 family, IL-33, has the ability to bind its precursor form to IL-1Rs and trigger signal transduction. Moreover, both IL-1β and IL-33 are also considered “dual-function” cytokines in that, in addition to binding to their respective cell surface receptors, their intracellular precursor forms have the ability to translocate to the nucleus and can influence subsequent downstream transcription (31, 32). In general, the nuclear function of IL-1α or IL-33 is transcription of pro-inflammatory genes. In contrast, the precursor forms of IL-1β and IL-18 do not bind to their respective receptors, are not active, requiring cleavage by either intracellular caspase-1 or extracellular neutrophilic proteases (6).

The biologic effects of IL-1 are regulated by naturally produced inhibitors, including IL-1Ra (IL-1F3), that binds to the IL-1RI and is specific for preventing the activity of IL-1α and IL-1β, without possessing any agonist function (6, 33). In addition, binding to the IL-1 receptor type II (IL-1RII), expressed mostly on macrophages, neutrophils, and B cells, does not result in productive signaling due to the lack of a cytoplasmic domain, for which docking of MyD88 cannot take place. IL-1RII binds IL-1β with a greater affinity than IL-1RI and works as a decoy receptor by sequestering IL-1β, thereby operating as a functional IL-1 antagonist. Because IL-1RAcP is recruited to the IL-1RII-IL-1β complex, the decoy receptor also serves to sequester the accessory receptor from participating in IL-1 signaling through IL-1RI (6). Finally, an additional tactic that IL-1Rs use to regulate the activity of IL-1 is by proteolytic cleavage of their extracellular domains. Shedding of IL-1RII results is the soluble form of IL-1RII (sIL-1RII) that has an increased affinity for IL-1β compared to IL-1α and IL-1Ra (34–38), thereby contributing to the antagonism of IL-1 by preferentially neutralizing IL-1β’s bioactivity. In addition, an alternate form of IL-1RAcP also exists that consists of only its extracellular domain; this soluble IL-1RAcP has the ability to associate with ligand-bound sIL-1RII, which results in an increased affinity of binding to both IL-1α and β, further establishing sIL-1RII as a potent inhibitor of IL-1 (39). Conversely, similar to its membrane bound form, sIL-1RI retains that ability to bind IL-1α and IL-1Ra with greater affinity than IL-1β, and can therefore be regarded as promoting a pro-inflammatory phenotype by sequestering IL-1Ra and limiting its anti-inflammatory effects on IL-1RI-bearing target cells, and by facilitating free IL-1β to bind to cell surface IL-1RI to promote pro-inflammatory immune responses (36, 38, 40, 41). Therefore, from a clinical perspective, the balance between IL-1 agonists, antagonists, and the amount of surface as well as soluble IL-1Rs affect the overall degree and severity of inflammation in several diseases, including IBD.

Gut mucosal inflammation is characterized by infiltration of neutrophils and mononuclear cells, which upon activation, are important sources of cytokines and other inflammatory mediators. IL-1α and IL-1β play key roles in intestinal inflammation, as they are produced early and induce the production of many other cytokines, amplifying their pro-inflammatory action (6). A marked increase in IL-1 production by isolated lamina propria mononuclear cells (LPMCs), most prominently from tissue histiocytes or macrophages, and by intestinal mucosal tissues has been reported in patients with active IBD by several groups (13, 42–44). Furthermore, tissue levels of IL-1 also closely correlate with the degree of observed mucosal inflammation and necrosis (9).

One of the earliest bodies of work dissecting the role of IL-1 in experimental colitis was performed using a rabbit immune complex-mediated model that possesses some features of UC (9, 12, 45, 46). Results from theses studies have provided insight into the bi-directional effects of an innate-type cytokine (i.e., IL-1). In this model, both IL-1α and IL-1β are increased in the inflamed intestinal tissues and display pro-inflammatory properties, as neutralization by either endogenous or exogenous IL-1Ra administration resulted in significant amelioration of colitis (12, 45, 46). Despite these findings, administration of recombinant IL-1β had a similar beneficial effect, indicating that IL-1β is necessary for mucosal protection and maintenance of homeostasis in this model (9). In fact, the currently accepted paradigm is that an imbalance of pro- and anti-inflammatory mediators, as exemplified by the IL-1/IL-1Ra system, is a key mechanism in the pathogenesis of IBD (47).

Interleukin-1 receptor antagonist, primarily produced by intestinal epithelial cells (IECs) and LPMCs within the gut mucosa (48), regulates the bioactivity of IL-1 and a marked decrease in the mucosal IL-1Ra/IL-1 ratio was found in both CD and UC patients when compared to control subjects (13). In this study, the IL-1Ra/IL-1 ratio correlated closely with the clinical severity of disease and was specific for IBD since this trend was not observed in patients with self-limiting colitis. Although the precise mechanism(s) as to why this imbalance occurs in IBD is not specifically known, several groups have reported an association between carriage of the IL-1RN allele 2 (IL-1RN^∗^2) of the IL-1Ra variable number of tandem repeats (VNTR) polymorphism and low production of IL-1Ra, as well as increased severity of disease in UC patients of several ethnic backgrounds (49–51). Finally, as indicated earlier, the expression and presence of cell surface and soluble IL-1Rs can affect the severity and overall disease phenotype that manifests in patients with IBD. In a study that surveyed circulating plasma and colonic tissue levels of IL-1α, IL-1β, IL-1Ra, sIL-1RI, and sIL-1RII from IBD patients and controls, it was found that sIL-1RI served as a systemic biomarker of disease activity in CD patients, while local shedding of the functional antagonist, sIL-1RII, was associated with decreased colonic inflammation in CD, but not in UC, patients (52).

**Figure 3:**
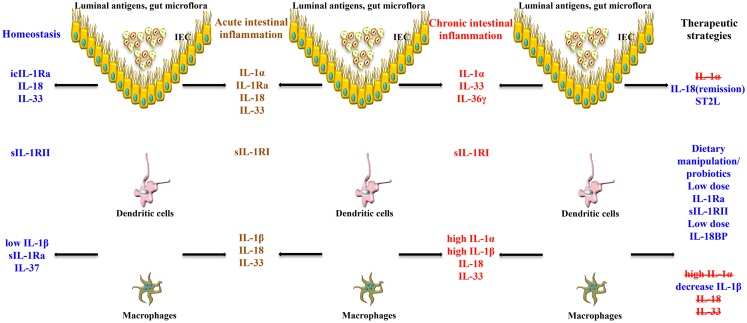
**Role of IL-1 family members in the gut mucosa and potential therapeutic strategies to restore intestinal homeostasis**. The effects of the same IL-1 family member can vary, and are often dichotomous, depending on the cell source, phase of disease (early vs. late), and inflammatory state (acute vs. chronic) of the host. IECs and APCs are critically involved in maintaining gut homeostasis, but also in triggering gut inflammation. icIL-1Ra, IL-18, and IL-33 released by IECs, together with sIL-1Ra, IL-37, and low levels of IL-1β produced by APCs, and sIL-1RII shed by macrophages, promote physiological inflammation, and homeostasis. Chronic intestinal inflammation results when this balance is disrupted, often changing the cellular sources and absolute levels of IL-1 family members. Based on this imbalance, therapeutic strategies aimed at modulating levels of IL-1 family members can be designed to reestablish gut health.

Taken together, the pathogenesis of chronic intestinal inflammation is characterized by a robust elevation of IL-1 family members promoting agonist activity, including IL-1α and IL-1β, whose primary source are LPMCs of myeloid lineage. A recent study, however, also provides evidence that during acute experimental colitis, IL-1α is potently produced by the intestinal epithelium (53). At the same time, production of IEC- and LP macrophage-derived IL-1Ra is not adequate to overcome the overwhelming pro-inflammatory effects of IL-1, resulting in perpetuation of chronic intestinal inflammation. This deficit in IL-1Ra can be due to carriage of a genetic polymorphism that infers low production, particularly in UC patients. Aside from IL-1 ligands, another facet of overall IL-1 biology to consider in a disease setting is the contribution of IL-1Rs. Within the gut mucosa, almost all cell types have the ability to respond to IL-1 ligands and express IL-1RI and II. The ability of these cells to shed soluble forms of IL-1Rs have indicated that sIL-1RI plasma levels may serve as a biomarker for disease activity and local sIL-1RII is associated with decreased colonic inflammation, specifically in CD patients. To date, however, a comprehensive study as to the precise distribution of IL-1Rs, including their co-receptors, their cellular sources, and potential trigger(s) to induce shedding during chronic intestinal inflammation, has not been performed. The results derived from these studies would provide critical information regarding the precise contribution of different IL-1R-bearing gut mucosal cell types during the course of disease, as well as aid in the design of more effective therapies to restore the IL-1/IL-1Ra imbalance (Figure 3).

### Pathogenic effects of IL-1α, IL-1β, and IL-1Ra in GI-related cancers

In addition to their contribution to chronic intestinal inflammation, IL-1 has also been implicated in tumorigenesis and tumor progression in the GI tract. Cancer cells can directly produce IL-1 or can induce cells within the tumor microenvironment to do so (54). IL-1β is upregulated in colon cancer, and patients with IL-1β-producing tumors generally possess a bad prognosis (55–57). The expression patterns of IL-1, in general, vary since it is expressed in either an autocrine or paracrine fashion (58). Co-culture studies on human melanocytic cells showed that IL-1α and IL-1 β exhibit autocrine behavior by stimulating tumor cells themselves to invade and proliferate, or exert paracrine effects on stromal cells in the microenvironment. The exact mechanism(s) by which IL-1 promotes tumor growth remain unclear, although it is believed to act primarily in an indirect fashion. In human colon cancer lines, IL-1 induces expression of metastatic genes, such as matrix metalloproteinases (MMPs), and stimulates nearby cells to produce angiogenic proteins and growth factors (59), including vascular endothelial growth factor (VEGF), IL-8, IL-6, tumor necrosis factor (TNF), and transforming growth factor (TGF)β (30, 60–62). Further studies in IL-1 transgenic mice demonstrate the necessity of IL-1 in tumor growth, metastasis, and angiogenesis (62, 63). Sawai et al. (64) evaluated the role of IL-1 in metastatic and non-metastatic human pancreatic cancer cell lines and showed that metastatic lines demonstrate increased IL-1RI expression compared to non-metastatic cell lines, and exposure to IL-1α results in increased α6- and β1-integrin subunit expression, whereas IL-1α exposure to non-metastatic lines has no effect. Additionally, IL-1α induces adhesion and invasion into laminin in human metastatic cell lines, but not in non-metastatic cell lines. This study highlights the importance of IL-1α for invasiveness and angiogenic properties *in vitro*, and confirms that only those cancer cell lines that show highly metastatic properties express IL-1α mRNA (65). These findings have also been confirmed for colon and gastric cancers. Human colon cancer-derived IL-1α induces angiogenesis by its action upon the microenvironment, and thereby contributes to metastasis (66). Along this same line, a significant correlation between IL-1α expression and metastasis in human gastric carcinomas has also been established (67, 68). Moreover, increased IL-1 production by gastric epithelial cells leads to gastric inflammation and the development of gastric dysplasia and cancer, as demonstrated in IL-1 transgenic mice (69). In fact, the administration of IL-1Ra has been proposed as a therapeutic regimen for different neoplasias (63).

Similar to IL-1, several lines of evidence point to the involvement of the another IL-1 family co-receptor member, single Ig IL-1R related molecule (SIGIRR), also known as Toll/IL-1R 8 (TIR8), in colitis-associated cancer in mice (70). SIGIRR/TIR8 is an orphan receptor that inhibits signaling from IL-1R/TLR complexes, possibly by trapping IL-1R-associated kinase (IRAK)-1 and TNFR-associated factor (TRAF)6 (71, 72), and is characterized by the presence of a single immunoglobulin domain in its extracellular region, a conserved TIR domain, and a 95-amino-acid long tail with inhibitory properties (73, 74) (Figure 1). SIGIRR/TIR8 is expressed in several tissues, especially in the digestive tract, and cell-type expression is particularly high in epithelial cells (74, 75). SIGIRR/TIR8 functions as a negative regulator for LPS and IL-1 signaling through its interaction with TLR4 and the IL-1R complex (76). Accordingly, there is evidence for a non-redundant regulatory role of this molecule in inflammation within the GI mucosa (75). *Tir8* deficient mice exhibit dramatic intestinal inflammation (colitis) in response to dextran sodium sulfate (DSS) administration in regard to weight loss, intestinal bleeding, and mortality, and show increased susceptibility to carcinogenesis in response to azoxymethane (AOM)/DSS administration (70). This increased susceptibility to colitis-associated cancer in *Tir8* deficient mice is linked to increased permeability and local production of prostaglandin E2 (PGE2), pro-inflammatory cytokines, and chemokines. In fact, colonic epithelial cells from *Tir8* deficient mice display commensal bacteria-dependent homeostatic defects, as shown by constitutive upregulation of pro-inflammatory genes, and increased inflammatory and tumorigenic responses to DSS and AOM/DSS challenge, respectively (77). As such, gut epithelial-specific expression of the *Tir8* transgene reduces colonic epithelial cell survival, abrogates the hypersensitivity of *Tir8* KO mice to DSS-induced colitis, and reduces AOM/DSS-induced tumorigenesis (77). These findings have been confirmed in *Apc^min/^(* mice, a spontaneous ileal polyposis model. Introduction of *Tir8* deficiency into the *Apc^min/^(* mice leads to increased loss of heterozygosity of *Apc* and colonic microadenoma formation. Importantly, the increased tumorigenesis in *Apc^min/^(/Tir8*^−/−^ mice is dependent on the presence of the commensal flora, underscoring the role of dysregulated commensal bacteria-TLR signaling in colonic tumor initiation (78).

**Figure 4:**
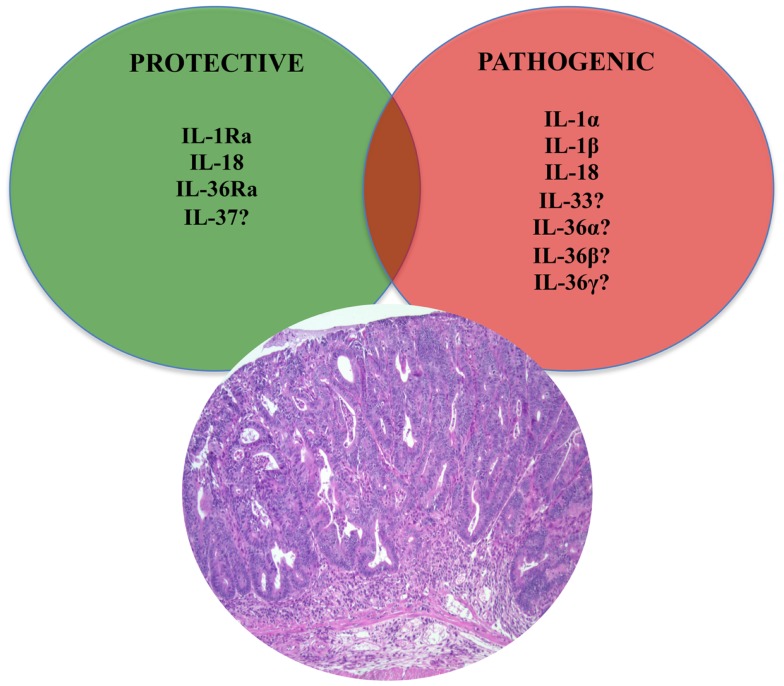
**Role of IL-1 family members in colon cancer**. Similar to chronic intestinal inflammation characteristic of IBD, an imbalance between protective and pathogenic IL-1 family members is also an important mechanism leading to intestinal tumorigenesis and the development of GI-related cancers. In fact, IL-1 cytokines play an important role in sustaining tumor growth by stimulating growth factor production and modulating host immune responses against tumor cells. While the roles of classic IL-1 family members, such as IL-1 and IL-18, have been firmly established, only speculation can be made on other, newer IL-1 family members, such as IL-33, IL-36, and IL-37.

The impact of the relationship between the gut microbiota and IL-1 family members on colitis-driven CRC also involves the inflammasome. Inflammasomes comprise, in essence, a multi-protein platform for the activation of inflammatory caspases, of which caspase-1 appears to play a dominant role (79). They include a sensor protein, an adaptor protein [apoptosis-associated speck-like protein (ASC) containing a caspase activation and recruitment domain (CARD)], and an inflammatory caspase. Sensor proteins belong to two families of proteins: the nucleotide-binding oligomerization domain (NOD)-like receptor (NLR) family and the pyrin and hemopoietic expression, interferon-inducibility, nuclear localization (HIN) domain-containing protein (PYHIN) family. Tight control of caspase-1 activation by inflammasomes, in particular of NOD-like receptor family pyrin domain-containing 3 (NLRP3, also referred to as Nalp3, CIAS1, or Cryopyrin), is critical since the processing and release of IL-1β and IL-18, as well as a subset of leaderless proteins that facilitate tissue repair, are directly regulated by caspase-1 (80). Homotypic interactions between the pyrin domain in the N-terminus of NLRP3 and the bipartite adaptor protein ASC (encoded by *Pycard*) bridge the association of caspase-1 to NLRP3 in the inflammasome. Mice lacking the inflammasome adaptor protein ASC and caspase-1 demonstrate increased disease outcome, morbidity, histopathology, and polyp formation in the AOM/DSS model of CRC (81). The increased tumor burden correlates with attenuated levels of IL-1β and IL-18 at the tumor site. In particular, leucine-rich-repeat-containing *Nlrp3*^−/−^ mice show an increase in acute and recurring colitis and colitis-associated cancer, although the disease outcome is less severe in *Nlrp*3^−/−^ mice than in *Pycard*^−/−^ or *Casp1*^−/−^ animals. No significant differences have been found in disease progression or outcome in NLR family CARD domain-containing protein 4 (*Nlrc4)*^−/−^ mice compared to similarly treated wildtype (WT) animals. Bone marrow reconstitution experiments show that *Nlrp3* gene expression and function in hematopoietic cells, rather than IECs or stromal cells, is responsible for protection against increased tumorigenesis (81). These data suggest that the inflammasome functions as an attenuator of colitis and colitis-driven CRC. Taken together, the imbalance of IL-1 agonists with IL-1 antagonists and their associated receptors/co-receptors within the GI tract may not be limited to promoting inflammatory processes, but may also be important in tumorigenesis and tumor progression (Figure 4). Re-establishing this balance may represent a new therapeutic target in the treatment of GI-related cancers.

### IL-18 in chronic intestinal inflammation

IL-18 (IL-1F4) was initially characterized as a novel IFNγ-inducing factor in mice infected with *Propionibacterium acnes* and subsequently challenged with a sublethal dose of LPS; as such, this factor was originally coined IFNγ inducing factor or IGIF (82). After cloning, IL-18 was shown to induce IFNγ in the presence of a mitogen or IL-2, and these effects were shown to be independent of IL-12 (83). IL-18 is widely expressed throughout various organ systems in the body and in cells of both hematopoietic and non-hematopoietic cell lineages (e.g., macrophages, dendritic cells, Kupffer cells, keratinocytes, osteoblasts, adrenal cortex cells, IECs, microglial cells, and synovial fibroblasts) (14, 84–90). Within the gut mucosa, IL-18 is primarily produced by IECs, tissue histiocytes (or macrophages), and dendritic cells (14, 15, 91). IL-18 exerts its biological effects through binding to the IL-18R complex, which is a heterodimer consisting of an α chain (IL-18Rα or IL-1R related protein 1, IL-1Rrp1), that is responsible for extracellular binding of IL-18, and a non-binding, signal-transducing β chain (IL-18Rβ or Accessory Protein Like, AcPL) (Figure 1). Both chains are members of the IL-1R family and are required for functional IL-18 signaling, that similar to IL-1, occurs through MyD88/IRAK, leading to the downstream activation of NF-κB (92–95) (Figure 2). The IL-18R complex is expressed on a variety of cell types, including T- and B-lymphocytes, macrophages, neutrophils, natural killer cells, endothelial cells, and smooth muscle cells (96–99). It can be upregulated on naïve T cells, Th1 cells, and B cells by IL-12 (93, 100). In contrast, T cell receptor ligation in the presence of IL-4 results in downregulation of the IL-18R complex (101). Although initially described as a Th1 polarizing cytokine, IL-18 has been shown to be a pleiotropic cytokine that can mediate both Th1- and Th2-driven immune responses (102, 103).

An additional family member that affects the overall bioactivity of IL-18 is the IL-18 binding protein (IL-18BP), a naturally occurring, soluble protein that effectively inhibits IL-18 by preventing its interaction with the endogenous receptor (Figure 1) (104). The human IL-18BP gene encodes at least four distinct isoforms (IL-18BPa-d), which are derived by alternative splicing (105). IL-18BP isoforms a and c neutralize the biological activity of IL-18, whereas b and d do not (106). The high affinity of IL-18 to the IL-18BP has a significant impact on its overall bioactivity and clinical relevance during disease states as ligand passing of IL-18 from the IL-18BP to its cell-bound receptor does not occur due to this unusually tight binding (104, 106, 107). Thus, the IL-18/IL-18BP system possesses several biological activities that underscore the potential for IL-18 to serve as a key mediator in the pathogenesis of several chronic inflammatory disorders, including IBD.

Our group and others were the first to report that IL-18 is upregulated in patients with IBD, particularly in CD (14, 15). IL-18 is present in the serum of CD patients, and bioactive IL-18 expression along with IL-18-induced cytokines are increased in mucosal biopsies of patients with IBD compared to controls, in involved vs. non-involved lesions, and in chronic advanced compared to early asymptomatic disease (14, 15, 91). Interestingly, immunohistochemistry (IHC) studies of colonic tissues derived from IBD patients and controls reveal a distinct pattern of IL-18 expression that may uncover potential IL-18-dependent mechanisms involved in maintaining gut health and in the pathogenesis of chronic intestinal inflammation (14) (Figure 3). In these studies, a dramatic shift in IL-18 expression is observed within the gut mucosa of CD patients as inflammation became more severe. In non-involved areas, IL-18 is immunolocalized almost exclusively to the epithelium, similar to that found in uninflamed tissues from the resected healthy margins of colon cancer patients. As disease severity increases, IL-18 expression switches from the epithelium to the lamina propria, specifically in cells morphologically consistent with tissue histiocytes/macrophages, wherein the most severe cases lack epithelial-derived IL-18. This trend appears to be specific for CD as IL-18 expression in UC patients is limited to the epithelium, independent of disease severity (14).

The IL-18 BP is also differentially expressed in intestinal tissues from IBD patients. Intestinal endothelial cells and macrophages are the major source of IL-18BP in the submucosa, and in CD, an increased number of IL-18BP-expressing macrophages and endothelial cells, specifically isoforms a, c, and d, has been detected (105). The presence of IL-18BP in CD lesions suggests neutralization of IL-18 activity, unless patients with active CD preferentially undergo differential splicing to produce more of the inactive isoforms (b and d) than the a and c bioactive isoforms. These patients would then have a reduced ability to regulate IL-18 activity during the course of the disease. In fact, free IL-18 is still observed in specimens from active CD and highlights the complexity of regulating bioactivity of IL-18. The importance of the IL-18BP in regulating IL-18 has also been reported in pediatric IBD patients, particularly in CD (108). IL-18BP does not sequester all free IL-18, which is increased not only local gut tissues, but also in the serum of children with active CD.

Although the majority of studies characterizing IL-18 and IL-18BP in IBD have been mostly descriptive in nature, they have laid the foundation that underscores the importance of the balance between IL-18 and IL-18BP in gut health and the pathogenesis of chronic intestinal inflammation, particularly in patients with CD. Similar to assessing global IL-1 bioactivity, expression of the IL-18R/co-receptor system on effector target cells should also be considered when evaluating the overall biological effects of IL-18. To date, a comprehensive study has not been performed to measure IL-18Rs and/or co-receptors in either CD or UC patients. However, polymorphisms in the IL-18 accessory protein (IL-18RAcP/IL-18RAcPL/IL-18Rβ), as well as IL-18, have been linked to IBD susceptibility (109–111). In fact, IL-18 expression is reportedly altered by a number of polymorphisms including three single-nucleotide polymorphisms (SNPs) in the IL-18 promoter at positions -137, -607, and -656, relative to the transcriptional start site (112). Transcription analysis of the first two polymorphisms showed that they cause altered transcription factor binding and gene expression (112). Similarly, SNP rs917997 correlates with altered expression of IL-18Rβ and is strongly associated with IBD and celiac disease (111, 113).

Animal models of IBD have provided a critical tool to mechanistically determine the potential role of IL-18 during the pathogenesis of colitis (120). Initial studies, in fact, support a pathogenic role for IL-18. In mice with either 2,4,6-trinitrobenzene sulfonic acid (TNBS)- or DSS-induced colitis, intestinal IL-18 levels of both macrophage and epithelial cell origin were found to be markedly elevated (114, 115). IL-18 expression, in co-operation with IL-12, leads to the expansion of Th1 CD4^+^ T cells (116) and production of the prototypic Th1 cytokines, IFN(, and TNF (117). Further evidence that IL-18 plays an important role in the chronic phase of intestinal inflammation was demonstrated using a T-cell dependent adoptive transfer model, wherein local administration of an adenovirus expressing anti-sense IL-18 mRNA had the ability to effectively treat colitis in recipient SCID mice (118). In fact, neutralization or targeted gene deletion of IL-18 results in amelioration of both chemically- and immunologically-mediated colitis (117–120), which may occur through a mechanism wherein local TNF production is dampened (114). Moreover, transgenic over-expression of IL-18 is associated with exacerbated colitis, which displays a marked infiltration of mucosal macrophages (121). The cellular re-distribution of IL-18, from IECs to gut mucosal macrophages, may be responsible for the pro-inflammatory role that IL-18 appears to play during chronic inflammatory responses within the gut mucosa. Using the SAMP1/YitFc (SAMP) model of spontaneous CD-like ileitis (122), our group previously reported that the mouse IL-18 gene is located within an interval on chromosome 9 that confers genetic susceptibility to disease in these mice (123). Similar to human CD, SAMP mice display a dramatic shift in the cellular source of IL-18 as disease becomes more severe, from IECs to LPMCs (124). The temporal and spatial expression of IL-18, in regard to the cellular source, as well as the presence or absence of specific IL-18R bearing cells, may explain the observed differential effects of IL-18 during the innate, early phases, vs. the later, chronic stages, of IBD. Together, these data indicate that IL-18 represents a central mediator in the pathogenesis of intestinal inflammation and is able to play very different roles during the inflammatory process depending on the host’s inflammatory state. As such, therapeutic strategies to alter IL-18 bioactivity need to be carefully addressed to determine the appropriate dose (low vs. high) and most beneficial time (early vs. late) to neutralize endogenously produced IL-18 during chronic inflammatory diseases, including IBD (Figure 3).

### IL-18 in inflammation-associated GI-related cancers

Aside from its established role in mucosal innate and adaptive immunity within the GI tract, IL-18 has also been identified as a mediator that both promotes and suppresses the process of oncogenesis (Figure 4). Although discussed in detail later in this review, IL-18’s protective effects include the ability to induce cell death and tumor regression through NK cell activation (125). In experimental cancer models, IL-18 expression in tumor cells has been shown to enhance both specific and non-specific anti-tumor immune responses (126, 127). On the other hand, IL-18 mRNA expression and serum levels correlate with the development and progression of gastric cancers (128), and may be associated with esophageal carcinoma. IL-18 upregulates expression of VEGF (129) and thrombospondin (130), suggesting its effectiveness in promoting angiogenesis. IL-18 also promotes metastasis by inducing cell adhesion molecules (131) and MMPs (132), while facilitating immune evasion by increasing the expression of Fas ligand on tumor cells (133). Similar to IBD, polymorphisms in the IL-18 promoter region are also strongly associated to GI-related cancers. In particular the rs917997 genotype appears to correlate with patient risk of reflux progressing to Barrett’s and esophagus adenocarcinoma (134, 135). IL-18 promoter polymorphisms are also associated with an increased risk for the development of gastric and colorectal cancers (136, 137).

## Protective Role of Classic IL-1 Family Members in Maintaining Intestinal Homeostasis and Gut Health

### Protective effects of IL-1α, IL-1β, and IL-1Ra

As previously mentioned, initial studies using a rabbit model of colitis revealed the potential dual role of IL-1 as a classic pro-inflammatory cytokine (12, 46, 138) as well as a mediator that has beneficial effects, particularly the IL-1β isoform, promoting gut mucosal protection (45). Interestingly, protection by IL-1β is only achieved with administration of low dose IL-1β, and only when given 24 h, but not 30 min, before the induction of colitis. Such protective effects of low dose IL-1 have also been shown in other disease models, such as arthritis (139) and sepsis (140). Similarly, in a mouse model of DSS-induced colitis, neutralization of IL-1 activity during the acute phase of disease was associated with exacerbated severity of inflammation and delayed recovery from injury (23). No effect was observed during the chronic stage of colitis, suggesting that IL-1 may have opposing effects during the progression of colitis by inferring protection during early, acute inflammation, but exerting more pro-inflammatory functions in later stages during the chronic phase of disease (Figure 3).

An alternative hypothesis to support the dichotomous role of IL-1 in IBD is that IL-1α and IL-1β possess opposing roles during the progression of chronic intestinal inflammation. In support of this concept, a recent study by Bersudsky et al. demonstrates that the precursor form of IL-1α, derived primarily from damaged IECs following DSS-induced colitis, can act as a classic alarmin by initiating and sustaining colitis, while IL-1α KO mice show little disease with increased recovery (53). Conversely, myeloid cell-derived IL-1β in the same colitic model induces the restitution and repair of IECs and improves gut barrier function during the recovery phase of acute inflammation. Furthermore, while specific blockade of IL-1α leads to amelioration of colitis, administration of IL-1Ra or anti-IL-1β antibodies do not effectively treat DSS colitis (53). Taken together, understanding the potential opposing roles of IL-1 agonists, such as IL-1α and IL-1β, during the initiation and progression of chronic intestinal inflammation, will shed further light on precise therapeutic modalities that will lead to more efficacious treatment of patients with IBD (Figure 3).

### IL-18-dependent protection during intestinal inflammation

Based on more recent studies, results point to the possibility of IL-18 possessing dichotomous roles during the progression of IBD, which may be related to phase of disease, as well as the cellular sources of both ligand and receptors/co-receptors (25). In fact, at the onset, or initiation of intestinal inflammation, IL-18 derived from IECs may exert a protective role, facilitating tissue repair and promoting mechanisms to induce homeostasis. In support of this concept is the observation that IL-18 and IL-18R KO mice are more susceptible to acute DSS colitis than their WT littermates (141). In addition, epithelial-derived IL-18 is critical for the protection from DSS colitis conferred by NLR-mediated signaling, as shown in studies utilizing mice deficient in *Nlrp3* (142).

In fact, similar to IL-1, emerging evidence highlights the control of IL-18 activation and the overall regulation of intestinal mucosal immune responses exerted by the inflammasome (80). As mentioned earlier, tight regulation of caspase-1 activation by inflammasomes is critical since the processing and release of IL-1β and IL-18 are directly regulated by caspase-1 (80). IL-18 is upregulated at the site of inflammation in DSS-exposed WT, but not in *Nlrp3*^−/−^, *Pycard*^−/−^, and *Casp1*^−/−^, mice (142). Nlrp3, Asc and Caspase-1/11 KO mice are also hyper-sensitive to acute DSS colitis, with low colonic IL-18 levels associated with disease susceptibility, while administration of exogenous IL-18 ameliorates colitis severity (142). Nevertheless, Bauer et al. (143) demonstrated that *Nlrp3* KO mice are protected from DSS-induced colitis, suggesting that DSS itself may activate the NLRP3 inflammasome. These results support that concept that different inflammasomes may exert differential and redundant effects on the development and progression of inflammation that may be additive or divergent, resulting in a hierarchical combinatorial net effect on intestinal inflammation (144). Thus, activation of a particular inflammasome in hematopoietic cells, such as dendritic cells and macrophages, may result in local release of IL-1β and/or IL-18 that induces inflammatory changes, such as secretion of IFNγ, while IL-18 secretion from IECs, through a different inflammasome, may play a local role in tissue regeneration and wound mucosal healing in response to injury. Such differential and cell-specific contributions of inflammasome signaling remain to be demonstrated experimentally. Moreover, inflammasomes are able to induce pyroptosis in damaged or infected IECs, which may affect tissue regeneration and consequently, the level of microbial influx into the LP and its effects on the severity of colitis (80). These effects may be induced by different inflammasomes and introduce a complex net effect based on temporal and microanatomical variations. However, mutations in the inflammasome pathway may also affect colitis differently, depending on the composition of the commensal microbiota that is present in the host since the inflammasome is a critical regulator of colonic microbial ecology (145). This observation also underscores the role of the commensal flora in intestinal immune homeostasis and further demonstrates the complexity of the gut mucosal immune system.

### Protective role of IL-18 in GI tumorigenesis

In contrast to its established, pathogenic role in tumorigenesis, IL-18 has been shown to represent a key protective cytokine in the development of inflammation-associated CRC using the AOM/DSS-induced model of colitis-associated cancer (146). An association between chronic inflammation and tumor development and progression is well established and as such, it is not surprising that a cytokine that has protective properties against inflammation can also reduce tumorigenesis associated with chronic inflammation. In fact, IL-18 and IL-18R KO mice are known to be highly susceptible to both DSS-induced colitis and colorectal tumorigenesis (147). In addition, MyD88 KO mice, which are defective in both IL-1β and IL-18 production, exhibit increased colonic epithelial proliferation, damage and colorectal tumorigenesis (147). Furthermore, administration of exogenous IL-18 can alleviate the severity of colitis and colitis-induced tumorigenesis in caspase-1/11 and Nlrp3 KO mice (148). In contrast, IL1R KO mice show equal numbers of colorectal tumors in the CRC AOM/DSS model, highlighting the unique and essential role of IL-18 during intestinal tumor progression (148). As such, and taking into consideration the previous discussion regarding IL-18’s pathogenic role in CRC, the contribution of IL-18 in tumorigenesis and the development of intestinal-specific cancer is clearly dichotomous. However, based on the current data, it appears that, similar to the role of IL-1 family members in intestinal inflammation, IL-18 primarily infers protection during early events leading to the development of GI cancers, including epithelial repair processes (147) and anti-tumor immune responses (126, 127), while during later stages, IL-18 supports events sustaining tumor growth [e.g., angiogenesis (129) and metastasis (130)].

### IL-37

IL-37 (IL-1F7) was first identified in 2000 and is one of the most recently characterized members of IL-1 family (149). In general, IL-37 has been shown to have potent anti-inflammatory properties and there is currently intense interest in elucidating its precise role in chronic intestinal inflammation and inflammation-associated CRC. Its relationship to IL-18 is that it binds to IL-18Rα, but unlike IL-18, it does not bind to the IL-18Rβ subunit or the accessory protein, IL-1RAcP (150–152) (Figures 1 and 2). Data, however, specifically investigating IL-37b, which is the most abundant form of IL-37 and the most studied, its binding to IL-18Rα, and whether IL-37 represents a competitive antagonist for IL-18 and its functions, remains unclear. An alternative hypothesis is that the IL-37b/IL-18Rα complex uses an accessory protein, such as SIGIRR/TIR8 (153), thereby activating a yet unknown anti-inflammatory pathway (Figure 1). It has also been suggested that IL-37 may bind weakly to the IL-18BP and render the IL-18Rβ useless for IL-18 by co-receptor competition (152) (Figure 1). In addition, recent studies have shown that the mature form of IL-37b may also translocate to the nucleus, similar to IL-1α and IL-33, and possess a regulatory role in gene transcription (154). At present, five splice variants (IL-37a-f) have been identified in humans; however, none of these variants are present in mice. Splice variant a, b, and c are expressed in lymph nodes, thymus, bone marrow, lung, testis, placenta uterus, skin, and colon; in addition, these variants are expressed in variety of immune cells, such as NK cells, monocytes, and stimulated B cells, while isoforms d and e are only expressed in testis and bone marrow (155). As mentioned earlier, IL-37b is the most abundant isoform and, relevant to the present review, is expressed in the cytoplasm of plasma cells in epithelial crypts, in the lamina propria of normal colon, and in the stroma of colon carcinomas. As with other IL-1 family members, IL-37 is synthesized as a precursor molecule that is cleaved by caspase-1 to its mature form (151).

In regard to its role in the pathogenesis of chronic intestinal inflammation and inflammation-associated CRC, very little has been reported at present (Table 1). *In vitro* studies on macrophages and epithelial cells overexpressing IL-37b, as well as *in vivo* experiments in transgenic mice overexpressing human IL-37b, show reduced DC activation and decreased production of pro-inflammatory and Th1/Th17 cytokines, including IL-1β, IL-6, IFNγ, and IL-17 following LPS stimulation. *In vivo* studies suggest that these effects may be mediated through the Smad3 pathway (156). In addition, IL-37b-tg mice exposed to DSS further upregulate IL-37b expression after epithelial injury and display a significant reduction in the severity of colitis compared to WT controls (16). IL-37 is also expressed in the colorectal carcinoma cell line, CCL-247, and in the stroma of colon cancer tumors, wherein IHC revealed intense staining in plasma cells of both normal and diseased colon, suggesting a potential role of IL-37 in antibody production, B-cell activation, and in colon tumorigenesis (151). Therefore, while initial reports indicate that IL-37 may play an anti-inflammatory role in acute colitis (Figure 3), further studies are warranted to elucidate the precise role in both chronic intestinal inflammation as well as inflammation-associated CRC (Figure 4).

## Dichotomous Role of IL-33, the Newest Member of the IL-1 Family, in Intestinal Inflammation and Mucosal Wound Healing

IL-33, also known as IL-1F11, is a protein with dual function that can act both as signaling cytokine as well as an intracellular nuclear factor (157) (Table 1). In the GI tract, IL-33 is primarily expressed in non-hematopoietic cells, including fibroblasts, adipocytes, smooth muscle cells, endothelial cells, and IECs (26, 158, 159), but is also present in cells of hematopoietic origin, particularly in restricted populations of professional antigen presenting cells, such as macrophages and DCs (26). IL-33 exerts its biological effects through binding to its receptor, IL-1 receptor-like 1 (IL1RL1), also known as ST2 (26, 28), and in the presence of IL-33, ST2 pairs with its co-receptor, IL-1RAcP, and signals through mitogen-activated protein kinase (MAPK)- and NF-κB-dependent pathways (26, 160) (Figure 2). Similar to IL-18Rα, the co-receptor SIGIRR/TIR8 can also dimerize with ST2 and likely acts as a negative regulator of the IL-33/ST2 signaling pathway, ultimately reducing IL-33’s biological effects (161) (Figure 1). To date, a very limited amount of information is available regarding the biologic and pathophysiologic relevance of IL-33 isoforms/splice variants, ST2 splice variants, and alternative ST2/SIGIRR signaling.

### IL-33 in maintaining gut homeostasis

In regard to its role in the GI tract, emerging evidence suggests that IL-33 plays a critical role in maintaining normal gut homeostasis. IL-33 enhances mucosal defenses against intestinal parasites and bacteria, as described for *Toxoplasma gondii* (162), *Pseudomonas aeruginosa* (163), and *Leptospira* (164) infection, indicating a primary role in mucosal protection. In addition, one of the earliest observations regarding the biological activity of IL-33 was its ability to promote epithelial proliferation and mucus production (26), which are obvious functions involved in epithelial restitution and repair, as well as overall mucosal wound healing and protection. Similar to IL-1α, increasing evidence also indicates that IL-33 can function as a prototypic “alarmin,” passively released upon cellular damage, stress, or necrosis, and able to serve as a danger signal/alarmin to alert the immune system of a local threat, such as trauma or infection (159, 165–167). In this setting, IL-33 has the ability to signal local, innate immune responses in an effort to mount an effective, physiological inflammatory reaction in order to restore normal gut homeostasis.

IL-33 has also been shown to activate mast cells, which are distributed throughout barrier tissues, such as the skin and mucosa, including the intraepithelial space of the intestine. Mast cells are classically considered important late-stage effector cells during Th2-associated immune responses, such as host responses against parasitic helminths in mucosal tissues (168). However, recent studies show that mast cells are able to initiate and orchestrate type 2 immunity against helminth infection through the regulation of tissue-derived cytokines. In fact, mast cell-deficient mouse strains and mice treated with the mast cell stabilizing agent, cromolyn sodium, show dramatically reduced Th2 priming and type 2 cytokine production and harbor an increased burden of parasites following infection with the GI helminthes, *Heligmosomoides polygyrus bakeri* and *Trichuris muris*. In addition, early production of the tissue-derived cytokines IL-25, IL-33, and thymic stromal lymphopoietin (TSLP), is significantly diminished in mast cell-deficient mice. Finally, repair of mast cell deficiency increases production of IL-25, IL-33, and TSLP, restores progenitor cell number and Th2 priming, and reduces intestinal parasite burden. These data reveal the important link between IL-33 and an innate IgE-independent role for mast cells in orchestrating type 2 immune responses. Mast cell degranulation, which is crucial for the activation of dendritic cells and recruitment of neutrophils and T cells to the site of infection (169–171), is also needed for the enhanced expression and production of the tissue-derived IL-25, IL-33, and TSLP, which are required for the optimal orchestration and priming of type 2 immunity (172, 173) and are obvious, apparent events important in intestinal mucosal protection against infection.

### IL-33/ST2 axis in IBD

In regard to chronic intestinal inflammation, it is now well established, and confirmed by several groups, that increased IL-33 expression is associated with IBD when compared to healthy controls, particularly in UC patients (17–20). In addition, a potential genetic predisposition to dysregulated IL-33/ST2 function may exist as a recent study describes the novel observation of association between the rs3939286 IL-33 polymorphism and IBD, and between the IL1RL1 rs13015714 and CD, in a well-characterized Italian cohort of adult and early onset IBD patients (155). The distribution of IL-33 expression in the gut mucosa is primarily localized to non-hematopoietic cells, particularly IECs (17, 18, 20) and myofibroblasts (19). In addition, *ex vivo* studies on isolated intestinal mucosal cell populations and immunolocalization on full-thickness intestinal tissues show that IL-33 is also expressed by a wide variety of cell types (17, 19, 22), such as fibroblasts, smooth muscle cells, endothelial cells (26, 174), and adipocytes (17, 158). In active UC, IL-33 is localized to, and potently expressed by, IECs, as well as infiltrating LPMCs, belonging to the monocyte/macrophage and B-cell lineages (17–19). It has also been originally reported by Kobori et al. (19), and later confirmed (22), that IL-33 is expressed in activated subepithelial myofibroblasts (SEMFs) situated below ulcerative lesions in UC, but not in CD, patients supporting a potential functional role for IL-33 in ulcer/wound healing, which may be different in UC compared to CD (Figure 3).

Similar to IL-33, its receptor, ST2, is also increased in the intestinal mucosa of IBD patients (17, 18). Importantly, the intestinal tissue expression pattern of ST2 is different in healthy mucosa compared to that found in chronically inflamed IBD patients, wherein ST2 is abundantly expressed in macroscopically non-inflamed colon epithelium, while during chronic inflammatory processes characterizing either UC or CD, its expression is lost/decreased and redistributed (28). This epithelial-derived tissue expression for ST2 appears to be IBD-specific since non-specific colitis (e.g., diverticulitis and infectious colitis) do not present with this same expression pattern (17). Taken together, considering the potential role of IL-33 in promoting mucosal protection, as well as its tissue distribution in IBD, it is tempting to speculate that the primary role for IL-33 is, in fact, to induce epithelial restitution and repair and mucosal healing (27). In addition, further analysis has shown that the ST2 variant for which expression is altered in the epithelium of IBD patients is ST2L, IL-33’s signaling transmembrane receptor (18, 28). As such, it is possible that impaired epithelial ST2L expression, specifically in IBD patients, may represent an inherent epithelial defect or a negative feedback response to chronic exposure of elevated IL-33 concentrations. One cannot rule out, however, that IL-33 may have pathogenic, as opposed to protective, effects by indirectly damaging or disrupting epithelial barrier function through, for example, recruitment of neutrophils and eosinophils, as well as consider its effects in mounting potent Th2, Th17, and potentiate Th1, immune responses that can amplify and sustain chronic intestinal inflammation. In fact, the dichotomous role of IL-33 has been best characterized in the intestine, where it can possess both protective and pro-inflammatory functions, depending upon the immunological status of the host and/or the type and phase of the ongoing inflammatory process (21, 28).

### Role of IL-33 in experimental models of acute colitis

Interestingly, investigation into the role of IL-33 in the development of intestinal inflammation using an acute model of DSS colitis has generated mixed results, and likely reflects the dichotomous roles of IL-33 in both inducing inflammation as well as promoting epithelial restitution/repair and mucosal healing. In fact, DSS administration to IL-33 deficient mice results in less severe colitis than in WT controls, with decreased granulocyte infiltration (175), while exogenous administration of IL-33 to DSS-treated mice further aggravates colitis and induces the influx of neutrophils (176), suggesting a pathogenic role of IL-33, at least in an acute inflammatory setting. Although it is unclear as to what factor(s) precisely regulate IL-33 in the gut, it has recently been shown that severe colonic inflammation with a marked increase in IL-33-producing macrophages results after DSS administration to mice expressing a truncated form of the receptor for TGFβ, supporting a pathogenic function for IL-33 during acute colitis and indicate a direct effect of TGFβ on macrophages to limit IL-33 expression (177). Imaeda et al. also reported an exacerbation of DSS-induced colitis upon treatment with IL-33, hypothesized to occur by IL-33-dependent induction of pathogenic Th2 cytokines; although in the same mice, IL-33 restores goblet cells that were found to be depleted in IL-33-untreated mice (178). In addition, during the recovery phase of DSS-induced colitis, while weight recovery is markedly delayed in IL-33 deficient mice, no significant difference in colonic inflammation is observed between these mice and WT littermates (175). The authors propose that in this particular model, IL-33 plays an important role in driving acute, innate immune responses, but is dispensable in the maintenance of chronic intestinal inflammation. Alternatively, the possibility exists that the delayed weight recovery observed in IL-33 deficient mice, but not in WT littermates, is due to the lack of IL-33-driven epithelial regeneration and restoration of barrier function leading to a dampened ability for mucosal healing.

In fact, as opposed to their results obtained from IL-33 treatment in acute DSS colitis, Groβ et al. showed that IL-33 administration during repeated, chronic cycling of DSS causes a reduction of colitis, suppresses IFNγ, and decreases bacterial translocation (176), supporting a protective role of IL-33 that the authors suggest may occur by switching from Th1- to Th2-driven immune responses. These results are supported by a recent study using the TNBS-induced model of colitis (179). Although the aforementioned study utilized an acute, 4-day protocol, exogenous administration of IL-33 was shown to ameliorate TNBS-induced colitis and induce the production of Th2-type cytokines (179). In addition, the protective effect of IL-33 was diminished after depletion of T-regulatory cells (T_regs_). The authors propose that, mechanistically, IL-33 has an indirect effect on the development of Foxp3^+^ T_regs_ by increasing the expression of epithelial-derived TSLP and retinoic acid, which promotes the activation of CD103^+^ DCs (180) and leads to the induction of Foxp3^+^ T_reg_ development (181). The ultimate IL-33-induced expansion of Foxp3^+^ T_regs_ facilitates the observed decrease in the severity of colitis.

### Role of IL-33 in experimental chronic intestinal inflammation

In SAMP mice, IL-33 expression patterns in the gut mucosa and within the systemic circulation of IBD patients were confirmed (17). IL-33 gut mucosal tissue levels in SAMP mice progressively increase over time and demonstrate a positive correlation with ileal inflammation, with epithelial cells exclusively expressing full-length IL-33 (17). Although the precise, mechanistic role of IL-33 has not yet been addressed in the SAMP model, preliminary studies blocking IL-33 signaling by administration of an antibody against ST2 indicate a pathogenic role during the chronic phase of disease development (182, 183). In fact, neutralization of IL-33 interferes with the massive influx of eosinophils into the gut mucosa (183) and potently decreases fibrosis and fibrogenic gene expression (182), characteristic of SAMP mice. Interestingly, although blockade of IL-33 has a significant effect on decreasing the overall severity of ileal inflammation in SAMP mice, the magnitude of this reduction is approximately 30%, which may reflect a need for optimizing treatment dosage or alternatively, represents an opposing effect of interfering with epithelial repair and mucosal healing. Investigation is further warranted to study the role of IL-33 during the early, acute phase of SAMP ileitis, as well as the specific role of epithelial-derived IL-33 and IL-33’s direct effects on the intestinal epithelium.

### IL-33 and ST2 in intestinal fibrosis

Although the role of IL-33 has not yet been fully investigated in the pathogenesis of intestinal fibrosis, several lines of evidence indicate that the IL-33/ST2 axis may represent an important mediator in this process. Within the gut mucosa, SEMFs have been reported as a primary source of IL-33, specifically in UC patients where they are situated below ulcerative mucosal lesions (19, 22). In fact, Sponheim et al. observed that a prominent feature of IBD-associated IL-33 expression is the accumulation of both fibroblasts and myofibroblasts in ulcerations of UC lesions (22). Although, the association and localization of IL-33-producing SEMFs with mucosal ulcerations suggests an important role in wound healing, one cannot rule out its potential role in gut-associated fibrosis, particularly in the setting of cycling of chronic tissue damage and repair, characteristic of IBD. Taken together, there is clear evidence of the IL-33/ST2 axis in maintaining normal gut homeostasis, particularly in promoting mucosal wound healing and repair. When dysregulated, this important ligand-binding pair can also play a critical role in the progression of chronic inflammation and fibrosis, leading to such GI-related disorders as IBD.

### Emerging role of the IL-33/ST2 axis in GI-related cancers

Finally, based on the established role of IL-1 family members in GI-related cancers, the possibility exists that IL-33 can likewise play an important role in GI-associated tumor formation. In fact, a recent study has reported elevated IL-33 levels in the serum of gastric cancer patients that correlates with several poor prognostic factors, including depth of invasion, distant metastasis, and advanced stage, but not with the classic tumor markers, CEA and CA 19-9 (184). Of note, however, no significant difference in IL-33 expression was found between four gastric cancer cell lines and the normal gastric cell line, GES-1, which may indicate that IL-33 expression can either be modulated by local environmental factors and/or produced by other cells responding to gastric cancer epithelial cells. As such, the initial observation of increased, circulating IL-33 levels in gastric cancer patients may be related to the progression of the cancer. In addition, based on IL-33’s ability to shift host immune responses to a Th2 phenotype, downregulation of tumor-specific immune responses can occur by inhibiting tumor antigen presentation (185, 186). From this point of view, IL-33 may represent one of the effective weapons tumor cells utilize in order to create an ideal environment to obtain, and maintain, optimal growth conditions, further supporting the role of the IL-33/ST2 axis in tumor formation and the progression of cancer (Figure 4).

### Potential contribution(s) of the IL-36-related cytokines in gut health and disease

In the last two decades, human genome sequence analysis has helped to identify new members of the IL-1 family. Three new members IL-36α, β, and γ, previously known as IL-1F6, IL-1F8, and IL-1F9, respectively, have been shown to bind to a heterodimeric receptor, IL-36R, also known as IL-1 receptor-related protein 2 (IL-1Rrp2), in a manner similar to the binding of IL-1α and IL-1β to IL-1RI. Consistent with the promiscuous nature of IL-1 family members, the IL-36 complex then recruits IL-1RAcP, thereby activating downstream NF-κB and MAPK pathways (187, 188). Interestingly, IL-36 family members also includes a receptor antagonist, IL-36Ra, similar to IL-1Ra, suggesting significant homology between these two IL-1 subfamilies (Figure 1).

At present, there are no known reports regarding the association between IL-36 and chronic intestinal inflammation, including IBD, as well as inflammation-associated CRC. Most of the published studies concerning IL-36 and disease pathogenesis come from either the psoriatic or pulmonary literature. In skin, all three IL-36 agonist ligands are highly expressed in psoriatic skin lesions (189–191). Johnston et al. has shown that TNF and IL-17 stimulation of human keratinocytes can induce IL-36, and IL-36 can, in turn, stimulate production of anti-microbial peptides and MMPs in human epidermal cells (192). Muhr et al. confirmed these findings and demonstrated that IL-17 potently induces greater amounts of IL-36 in keratinocytes obtained from psoriatic patients compared to healthy controls (193). Others have described increased expression of TNF and IL-6 in IL-36-stimulated keratinocytes, suggesting a mutual regulation of these inflammatory mediators (194). Recent studies have also supported an important role of IL-36γ in various lung pathologies. IL-36γ expression is reported to be increased in mice after allergen challenge, and intratracheal administration of IL-36γ leads to airway hyper-responsiveness, neutrophil accumulation and pro-inflammatory cytokine production (195–197). In addition, IL-36 signaling promotes Th1 polarization of naïve CD4^+^ T cells (198) and induction of Th17 immune response in lung disease (196, 199). Finally, increased IL-36α expression was reported in eosinophilic esophagitis, indicating a possible role of IL-36 in Th2-type immune responses (200). Taken together, these data imply an important pro-inflammatory role for IL-36 ligands in chronic immune disorders, although it is unclear at present whether IL-36 is prone to promoting Th1, Th2, and/or Th17 immunity and whether, like other IL-1 family members, IL-36 may possess dichotomous functions in the setting of health and disease states. In addition to the three described IL-36 agonists, the IL-36Ra and IL-38, previously known as IL-1F5 and IL-1F10, respectively, also bind to the IL-36R; however, differently from IL-36α, β, and γ, IL-36Ra and IL-38 both serve as antagonists for the biological activities of IL-36 (187, 188, 190, 201). Interestingly, IL-36Ra has been shown to possess an anti-inflammatory effect localized to the brain and mediated through a unique TIR8/SIGIRR-dependent pathway (202).

On the basis of the limited availability of published data and preliminary findings, IL-36 may potentially play an important role in chronic inflammatory disorders, including IBD. Investigation into the role of the IL-36 family of cytokines in chronic intestinal inflammation and inflammation-associated CRC, in fact, is an active area of research that may uncover further pathogenic mechanism(s) involved in GI-related pathologies and may provide the foundation for IL-36 to serve as a potential therapeutic target in the near future.

## Conclusion

**Figure 5:**
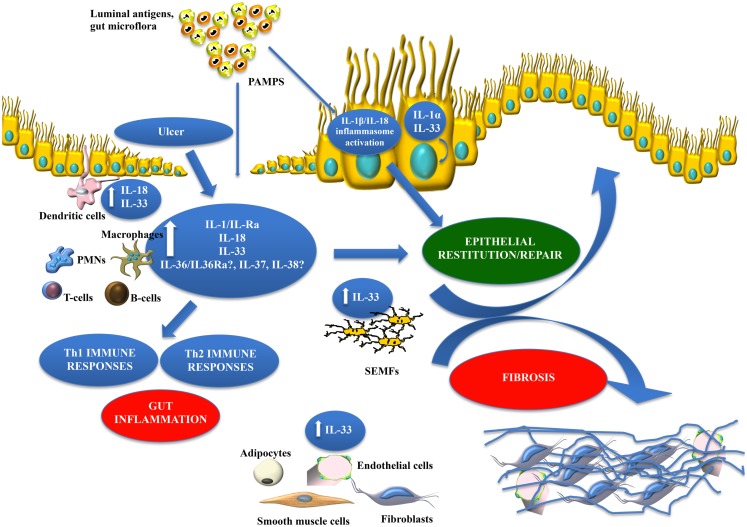
**Working hypothesis summarizing opposing functions of IL-1 family members within the gut mucosa**. The balance between pro-inflammatory and protective cytokines is crucial for the maintenance of gut homeostasis. Damage to the epithelium (e.g., ulcer formation) and other pro-inflammatory stimuli, including PAMPs derived from luminal antigens and the local intestinal microflora, induce the expression of IL-1 family members that are subsequently released by necrotic IECs as potential alarmins (e.g., IL-33 and IL-1α). Depending on the cellular source and presence of receptor-bearing effector cells, IL-18 can possess very different functions within the gut mucosa. IL-33 may also act on various immune cell populations, including macrophages, and T- and B-cells, eliciting a pro-inflammatory response and promoting Th2 immunity. Concomitantly, IL-33 can also induce epithelial proliferation and repair, and overall wound healing by acting directly or indirectly on IECs and SEMFs. Alternatively, chronic mucosal damage, granulomatous inflammation, and dysregulated activation of mesenchymal cells, such as SEMFs and fibroblasts, can lead to fibrosis and the formation of intestinal fibrotic lesions. Therapeutic interventions should consider all of the aforementioned processes and whether targeting specific IL-1 family members may be more efficacious during active disease vs. maintaining remission.

The present review provides evidence that members of the IL-1 family of cytokines possess dichotomous, often opposing functions in both the maintenance of normal gut homeostasis and in the pathogenesis of chronic intestinal inflammation and inflammation-associated CRC. We hypothesize that their effects vary, depending on the phase of disease (early vs. late), as well as the inflammatory state (acute vs. chronic) of the host. In general, early activation of the intestinal epithelium by pathogenic organisms and/or other noxious environmental antigens elicits the production of epithelial-derived IL-1 family members, including intracellular (ic)IL-1Ra, IL-1α, IL-18, and IL-33. Epithelial disruption often occurs, facilitating translocation of luminal bacterial products and the recruitment of innate immune cells, primarily neutrophils, and macrophages that are also a potent source of secreted (s)IL-1Ra, IL-1β, IL-18, and IL-33. Normally, early expression of these mediators function to dampen acute inflammation and promote epithelial repair and restitution, with the end goal of limiting gut mucosal damage and restoring intestinal homeostasis. Under conditions of either uncontrolled and/or persistent inflammation (e.g., as a result of innate immune dysfunction or host genetic predisposition), infiltration of adaptive immune cells bearing various IL-1R family members occurs during the later phases of inflammation, making available an effector population able to respond to IL-1-like ligands. For example, the presence of naïve CD4^+^ T cells expressing the IL-18R have the ability to respond to IL-18, and in combination with IL-12, represents one of the most potent stimuli for IFNγ production and Th1 polarized effector responses, thereby promoting chronic Th1-mediated inflammation. Similar effects can occur upon IL-33 stimulation of naïve CD4^+^ T cells, but in this case, a robust Th2 immune response results. Furthermore, several levels of regulation exist within each subfamily of IL-1 family members, often including the presence of several agonist isoforms (both precursor and mature, cleaved forms), receptor antagonists, as well as soluble and cell-bound decoy receptors. In addition, the promiscuity of IL-1 family ligands with both binding receptors as well as recruited accessory proteins, instills yet another level of regulation that should be considered when determining the overall biological effects of a specific IL-1 family member agonist. In fact, IL-1 family members cannot be considered in isolation, but with other IL-1-related proteins that can influence their overall interactive effects. An imbalance in the equilibrium between IL-1 family components, dependent on prevalent isoform and receptor binding domain/accessory protein present on effector cells, maybe responsible for either driving pathogenic events, including chronic intestinal inflammation, fibrosis, and CRC, or for promoting protection by inducing epithelial repair, mucosal wound healing, and restoration of gut homeostasis (summarized in Figure 5). Based on this new information and the emerging concept that IL-1 family members can possess opposing role in gut health and disease, novel pathogenic hypotheses can be formed that have important translational implications in regard to the prevention and treatment of chronic intestinal inflammation, including CD and UC, and CRC.

## Conflict of Interest Statement

The authors declare that the research was conducted in the absence of any commercial or financial relationships that could be construed as a potential conflict of interest.
